# Supplementary material for: Complex‐centric proteome profiling by SEC‐SWATH‐MS
Source: Mol Syst Biol. 2019 Jan 14;15(1):e8438. doi: 10.15252/msb.20188438 (PMC6346213; doi:10.15252/msb.20188438)
Supplement: Supplementary file 8 — Dataset EV7 [file MSB-15-e8438-s008.zip › feature_plots_string/Q86TM6.pdf]

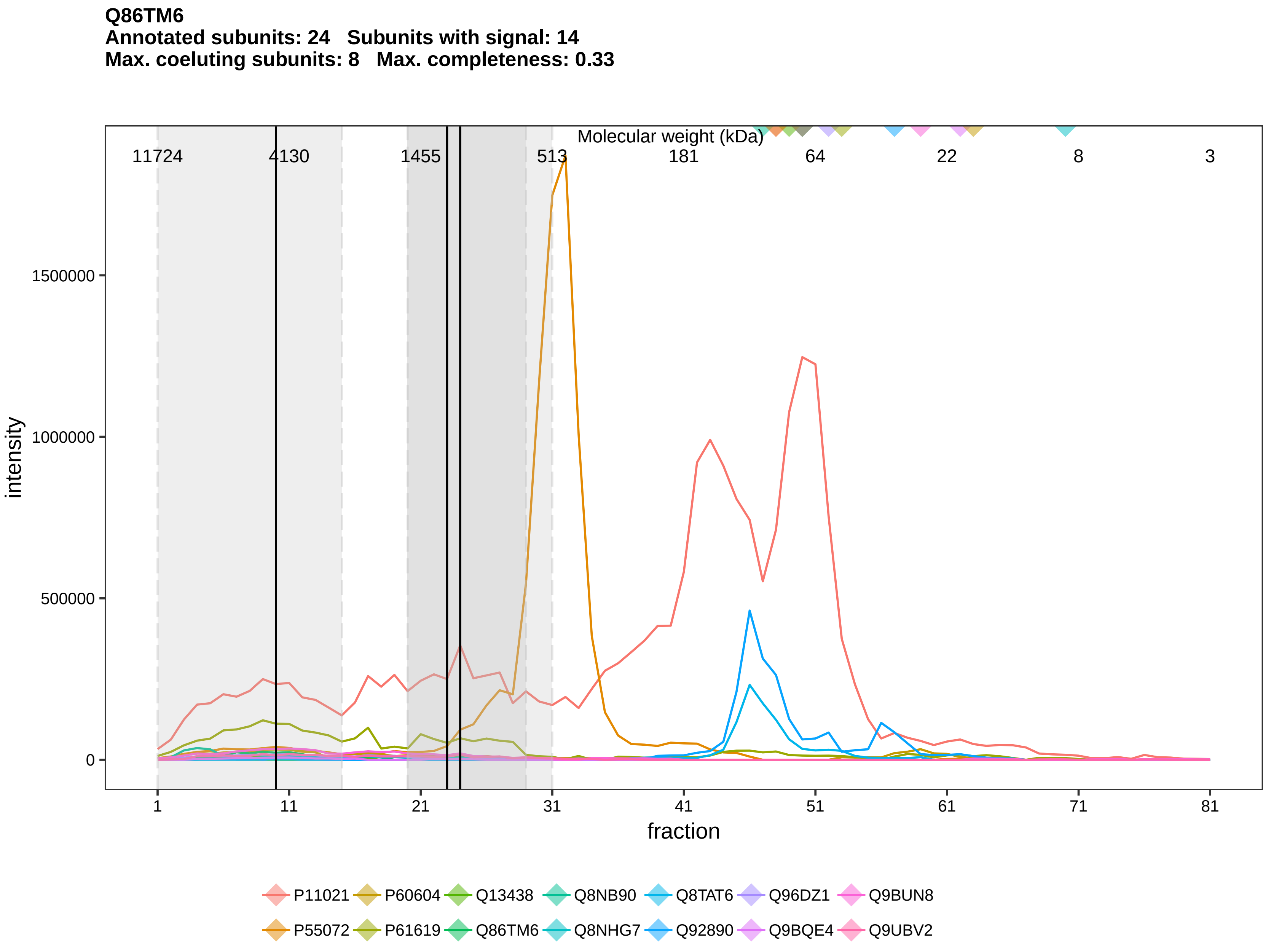
Q86TM6
Annotated subunits: 24 Subunits with signal: 14
Max. coeluting subunits: 8 Max. completeness: 0.33
Molecular weight (kDa)
11724
4130
1455
513
181
64
22
8
3
intensity
1500000
1000000
500000
0
fraction
1
11
21
31
41
51
61
71
81
P11021
P60604
Q13438
Q8NB90
Q8TAT6
Q96DZ1
Q9BUN8
P55072
P61619
Q86TM6
Q8NHG7
Q92890
Q9BQE4
Q9UBV2
